# Odontogenic Cutaneous Fistula: First Dermoscopic Description and a Practical Management Framework

**DOI:** 10.7759/cureus.92791

**Published:** 2025-09-20

**Authors:** Jesús Iván Martínez-Ortega, Alejandra Nicole Macias Quiroga

**Affiliations:** 1 Histology, Autonomous University of Nuevo Leon, San Nicolás de los Garza, MEX; 2 Dermatology, Dermatology Institute of Jalisco, Zapopan, MEX; 3 Dermatology, Hospital Obrero No. 1, La Paz, BOL

**Keywords:** dental infection, dermoscopy, drainage, facial abscess, fistula, odontogenic cutaneous fistula, odontogenic cysts, osteomyelitis, primary health care

## Abstract

Odontogenic cutaneous fistula (OCF) is an uncommon manifestation of dental infection that is often underrecognized because of its variable morphology and the absence of obvious dental symptoms. Misdiagnosis may lead to unnecessary tests and treatments, whereas a permanent cure depends on the elimination of the offending tooth. We report the case of a 15-year-old female who presented with progressive swelling of the right cheek and fever. Ultrasound revealed a 21.8-cc abscess, which was managed with surgical drainage and a five-day course of amoxicillin-clavulanate. Dermoscopy showed radiating white streaks surrounding the ulcer, peripheral scaling, central erosions, and serpentine vessels. These findings likely correspond to fibrosis around the fistulous tract and, to our knowledge, represent the first dermoscopic description of OCF. The patient was lost to follow-up before dental imaging could confirm the tract and identify the causative tooth. OCF presentations span a spectrum, from acute abscess-driven lesions in younger patients to chronic cases associated with osteomyelitis in the elderly, with most cases recognized in routine dermatology or dental consultations. While the majority are not abscess-associated and can be managed directly with endodontic or surgical therapy, acute cases require urgent drainage and antibiotics before definitive dental treatment. We illustrate a practical three-step framework: assessment of severity and location, stabilization of acute presentations, and elimination of the source tooth, which emphasizes anatomical location and chronicity over morphology and may improve early recognition and effective management.

## Introduction

Odontogenic cutaneous fistula (OCF) is an uncommon skin manifestation of chronic dental infection in which a periapical or periodontal focus tracks through bone and soft tissue to drain via the skin.

OCF is frequently underdiagnosed because its cutaneous morphology is polymorphic (nodule, abscess, cyst, dimpled scar) and dental symptoms may be absent or unrecognized. In the largest retrospective series (75 patients) from a tertiary dermatology center, the diagnosis of OCF was initially suspected in only 51%, meaning half were misinterpreted as other dermatoses and subjected to superfluous tests or treatments [1].

This occurred even within a center co-located with dental specialists, highlighting a diagnostic blind spot at the interface of dermatology, dentistry, and primary care [1]. In an era of increasing specialization, clinicians often approach conditions primarily through the lens of their own discipline, leading to delays in recognizing diseases that straddle multiple domains. Importantly, early recognition is essential: initial evaluation and treatment are often provided by dermatologists or primary care physicians, but definitive cure requires elimination of the dental source, usually through endodontic therapy or extraction by dental surgeons [1,2].

## Case presentation

A 15-year-old female presented with a two-week history of progressive swelling of the right cheek, accompanied by intermittent fever up to 38.5°C.

**Figure 1:**
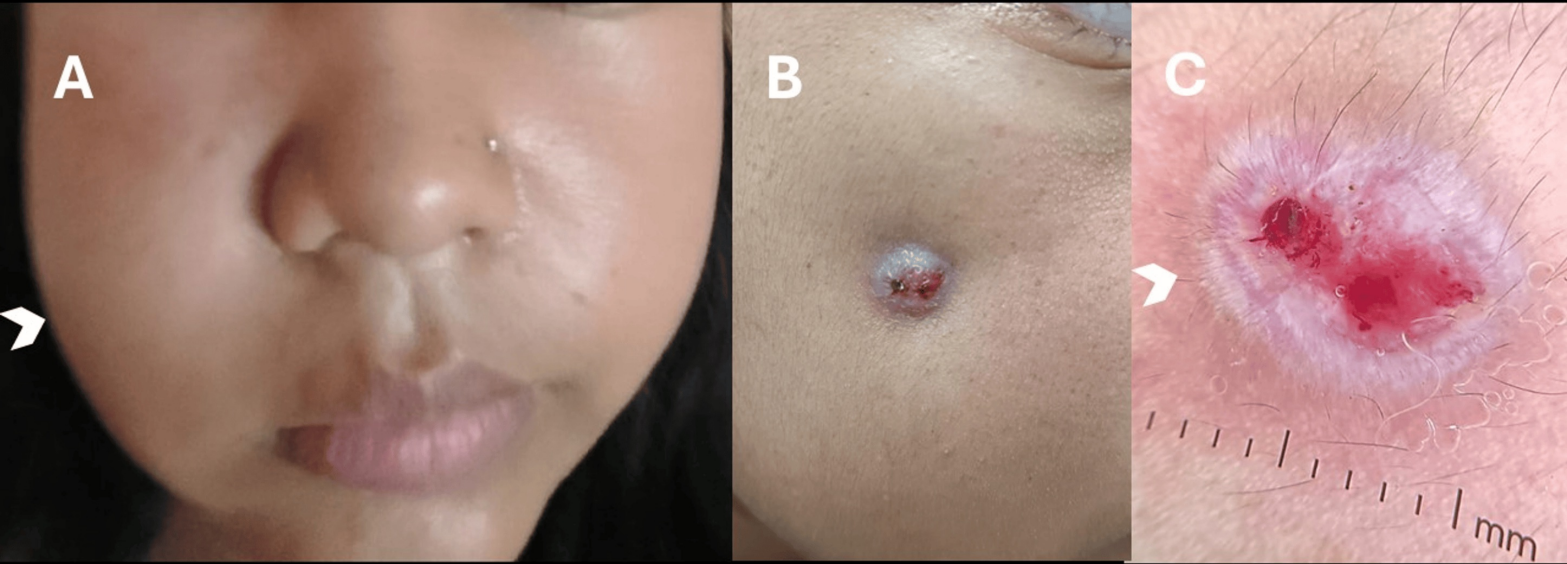
Clinical and dermoscopic features of odontogenic cutaneous fistula in a 15-year-old female (A) Right cheek swelling with facial asymmetry (arrowhead at lesion site). (B) Crateriform 7 × 4 mm nodule with central depression and retraction on the right cheek. (C) Polarized contact dermoscopy (DermLite 4, DermLite, Aliso Viejo, CA, 10×magnification) showing radiating white streaks, peripheral scaling, central erosions, and serpentine vessels (arrowhead).

On examination, a 9 × 5 mm crateriform nodule was present on the right cheek. Palpation demonstrated induration with focal fluctuance, consistent with a localized abscess. Gentle pressure expressed purulent drainage overlying the swollen area (Figures 1A-1B).

Intraoral inspection revealed caries involving the three right maxillary molars; however, vitality testing and radiographic evaluation were not performed. Based on lesion topography, the right maxillary first molar was considered the most likely source.

Polarized contact dermoscopy demonstrated radiating white streaks, peripheral scaling, central erosions, and serpentine vessels, findings consistent with fibrosis and vascular changes surrounding a probable fistulous tract (Figure 1C).

**Figure 2:**
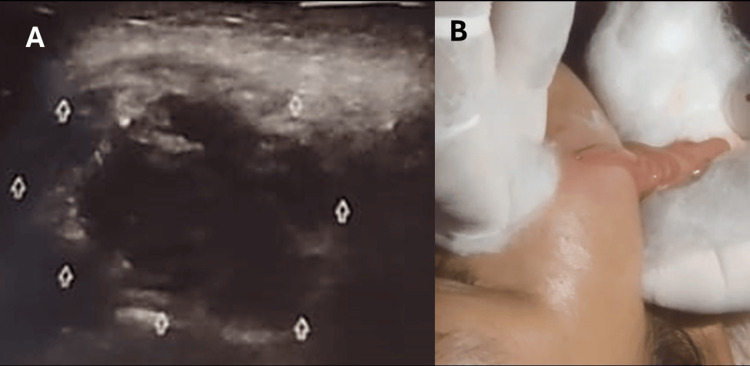
Ultrasound and surgical drainage of the facial abscess (A) Point-of-care ultrasound revealing a heterogeneous fluid collection (21.8 cc) in the right cheek. (B) Intraoperative image during incision and drainage of the abscess cavity.

Point-of-care ultrasound identified a heterogeneous 21.8-cc fluid collection in the right cheek (Figure 2A). No fistulous tract was detected. Urgent incision and drainage were performed via the skin surface, evacuating purulent material. During the procedure, efflux extending inward toward the buccal pad was observed, raising suspicion of a fistulous connection (Figure 2B).

The patient received a five-day course of oral amoxicillin-clavulanate (875/125 mg every 12 hours) and subsequently improved. Unfortunately, she was lost to follow-up before dental imaging (periapical radiograph or cone-beam CT) could confirm the fistulous tract and identify the specific causative tooth. As a result, while the acute abscess was successfully drained, the underlying odontogenic source could not be definitively identified or treated, limiting the possibility of permanent cure. The case, therefore, represents a presumptive OCF, supported by clinical, intraoral, dermoscopic, and intraoperative findings.

## Discussion

OCF is often overlooked because its cutaneous morphology is nonspecific. Lesions may appear as nodules, abscesses, cysts, draining sinuses, or dimpling scars. In the Korean series, morphology was highly variable: dimpling (41%), nodules (32%), abscesses (21%), and cysts (6%). Therefore, morphology alone is unreliable for diagnosis [2]. In the same Korean cohort, 27/33 patients were referred after one or more recurrences following misdiagnosis or inappropriate therapy, underscoring the challenge of under-recognition. Notably, buccal cheek involvement occurred in ~14.7% of cases, an uncommon location compared to the mandibular body and chin, which account for nearly half of presentations [2].

In practice, the most valuable clues to diagnosis are anatomical location and chronicity of the lesion, particularly when accompanied by evidence of dental pathology. In our patient, the presence of caries in the right maxillary molars provided an important contextual clue, even though radiographic confirmation was not obtained. Recurrent draining lesions situated overlying the teeth, most often at the mandibular angle or chin, and less frequently on the cheek or nasolabial fold, should prompt consideration of OCF [1,2].

The diagnostic delay is further explained by the increasing fragmentation of medical practice: dermatologists, dentists, surgeons, and infectious disease specialists each approach the lesion from within their own framework. In conditions like OCF, which lie at the interface of dermatology and dentistry, this leads to underrecognition and mismanagement. In the series by Guevara-Gutiérrez et al. [2], nearly half of the cases underwent unnecessary investigations (mycological studies, chest radiographs, biopsies), while others were misdiagnosed as tuberculosis, actinomycosis, or neoplasms [1]. Similar patterns were seen in Lee et al., where most patients had recurrence due to ineffective treatment before referral [1].

Our patient is also remarkable for her young age (15 years). Although earlier literature suggested OCF was more common in children and adolescents, large modern series actually show a predominance in adults (mean age 40-45 years), suggesting that pediatric cases are less frequent than previously assumed [1,2]. The buccal cheek, reported in 14.7% of cases by Lee et al., and the cheek location in our patient, are considered uncommon compared to the mandibular body and chin, which account for up to 50% of cases [1].

Dermoscopy revealed radiating white streaks encircling the ulcer features, likely corresponding to fibrotic stroma around the fistulous tract. Similar shiny white structures (also described as crystalline, chrysalis, or orthogonal streaks) have been reported in various lesions, including scars, dermatofibromas, basal cell carcinoma, and melanoma. These structures are visible only with polarized-light dermoscopy and are attributed to the birefringent properties of collagen bundles rapidly randomizing polarized light [3,4]. To our knowledge, dermoscopic features of an OCF have not been described previously. The observed white streaks here may represent a novel pattern - perhaps best termed a “radiating fibrotic corona” - that reflects fibrosis encircling a fistulous tract

The differential diagnosis of OCF is broad because its cutaneous morphology is nonspecific. Infectious mimickers include actinomycosis, scrofuloderma, and deep fungal infections, while noninfectious possibilities range from epidermal inclusion cysts and foreign-body granulomas to neoplasms such as basal cell carcinoma, cutaneous lymphoma, or even metastatic deposits. What ultimately distinguishes OCF is not morphology but context: the lesion’s proximity to teeth, its recurrent drainage, and radiologic correlation. Orthopantomography or periapical radiographs and, when necessary, gutta-percha tract tracing, provide the decisive link to a dental focus and allow definitive diagnosis [1,2].

The spectrum of OCFs extends from acute lesions in younger patients to chronic presentations in the elderly. In the Korean series, the longest disease duration was observed in an elderly patient who evolved for 144 weeks before diagnosis, ultimately attributable to chronic osteomyelitis of the mandible [2]. At the other end of the spectrum, acute lesions in children or adolescents may reflect an abscess-driven process, as illustrated by our case. These extremes demand different clinical reasoning: whereas elderly patients with long-standing lesions warrant evaluation for osteomyelitis, acute swelling in younger patients should raise suspicion of an odontogenic abscess. Most OCFs, however, fall within the intermediate range and are diagnosed in the setting of routine primary care, dental, or dermatology consultations, where they can be readily confirmed and treated with standard endodontic or surgical therapy [1,2].

The differential diagnosis of OCF is broad because its morphology is nonspecific. Infectious mimickers include actinomycosis, scrofuloderma, and deep fungal infections, while noninfectious possibilities include epidermal cysts, foreign-body granulomas, or neoplasms [1,2]. In endemic regions, tropical ulcers, particularly cutaneous leishmaniasis, should also be considered, since they may rarely fistulize [5]. However, in our patient, the presence of a large abscess and intraoral caries favored an odontogenic origin.

Importantly, the majority of OCFs are not associated with acute abscesses and therefore do not require urgent drainage. In such cases, definitive management consists of identifying and eliminating the dental source, either by root canal therapy or extraction, with antibiotics reserved for patients who exhibit systemic features [1,2]. When an abscess is present, particularly in anatomically sensitive regions, the acute infection must first be controlled before proceeding to definitive dental treatment. Building on established principles of odontogenic infection control, we illustrate a practical three-step framework, adapted from established principles of odontogenic infection control, which may aid clinicians when encountering OCF. First, assess severity and location, distinguishing localized, stable lesions from those with systemic red flags such as fever or leukocytosis, or with clinical evidence of high-risk fascial space involvement, including facial swelling, trismus, dysphagia, or airway compromise. Second, stabilize the acute setting when required, performing urgent incision and drainage and initiating empiric antibiotics, and escalating to multidisciplinary care if red flags such as rapidly progressive facial swelling, trismus, dysphagia, or airway compromise are present. Third, eliminate the source tooth, either after stabilization in abscess-driven cases or directly in non-abscess presentations, through endodontic therapy or extraction. This simplified framework integrates the core principles of odontogenic infection management into a clinically useful dichotomy: most cases can proceed directly to definitive dental treatment, whereas abscess-driven presentations require an initial stabilization step before permanent cure can be achieved (Table 1) [6-8].

**Table 1:** Proposed three-step management framework for odontogenic cutaneous fistula (OCF) The framework emphasizes that diagnosis depends more on anatomical context than morphology, and that permanent cure requires elimination of the odontogenic source. While antibiotics and drainage may stabilize acute presentations, definitive management relies on endodontic therapy or extraction of the causative tooth.

Step	Action	Key considerations
1. Assess severity and location	Evaluate lesion topography and systemic status	Morphology is unreliable (nodule, abscess, cyst, scar). Key diagnostic clues: proximity to teeth, chronicity, dental history. Identify systemic red flags: fever, leukocytosis, trismus, dysphagia, airway compromise, rapidly progressive swelling.
2. Stabilize acute presentation (if present)	Control infection and prevent complications	Perform urgent incision and drainage. Initiate empiric antibiotics. Escalate to multidisciplinary care if fascial space involvement or airway risk.
3. Eliminate the source tooth	Definitive management	Perform endodontic therapy or extraction of the causative tooth. Permanent cure requires elimination of the odontogenic focus. Antibiotics alone may not be sufficient for definitive cure.

It should be noted that this stepwise approach is intended for primary care or dermatology consultations where odontogenic infection may first be suspected; in dedicated dental or maxillofacial settings equipped for surgical intervention, the pathway would naturally be adapted, and escalation to a multidisciplinary unit may not be required since definitive surgical management can be performed directly.

This case highlights several important limitations. The patient was lost to follow-up before dental imaging (periapical radiograph or cone-beam CT) could confirm the fistulous tract and identify the causative tooth. This absence of definitive radiographic confirmation is a significant limitation, as it precludes absolute diagnostic certainty and prevents definitive treatment. In addition, the description of lesion size and palpation, while provided, remains less detailed than would be ideal for reproducibility. Finally, as this is a single case, the generalizability of the dermoscopic features observed (“radiating fibrotic corona”) remains to be validated in larger cohorts.

## Conclusions

OCF remains an underrecognized condition that is frequently misdiagnosed, even in specialized settings. Most cases can be cured with definitive dental treatment; however, acute abscess-driven presentations require an initial stabilization step, including drainage and antibiotics, before source control can be achieved. Emphasizing anatomical location and chronicity over morphology, and applying a systematic three-step framework: assessment of severity, stabilization of acute cases, and elimination of the source tooth, may improve diagnostic accuracy, reduce unnecessary investigations, and facilitate definitive cure.
